# Infrared thermography can detect previsual bacterial growth in a laboratory setting via metabolic heat detection

**DOI:** 10.1111/jam.15218

**Published:** 2021-07-24

**Authors:** Ben Hunt, Reza Saatchi, Melissa M. Lacey

**Affiliations:** ^1^ Biomolecular Sciences Research Centre Sheffield Hallam University Sheffield UK; ^2^ Centre for Automation and Robotics Research Materials and Engineering Research Institute Sheffield Hallam University Sheffield UK

**Keywords:** detection, *E. coli*, metabolism, Staphylococci, thermal processes

## Abstract

**Aims:**

Detection of bacterial contamination in healthcare and industry takes many hours if not days. Thermal imaging, the measurement of heat by an infrared camera, was investigated as a potential noninvasive method of detecting bacterial growth.

**Methods and Results:**

Infrared thermography can detect the presence of *Escherichia coli* and *Staphylococcus aureus* on solid growth media by an increase in temperature before they are visually observable. A heat decrease is observed after treatment with ultraviolet light and heat increased after incubation with dinitrophenol.

**Conclusions:**

Infrared thermography can detect early growth of bacteria before they are detectable by other microbiology‐based method. The heat observed is due to the cells being viable and metabolically active, as cells killed with ultraviolet light exhibit reduced increase in temperature and treatment with dinitrophenol increases heat detected.

**Significance and Impact of the Study:**

Infrared thermography detects bacterial growth without the need for specialized temperature control facilities. The method is statistically robust and can be undertaken in situ, thus is highly versatile. These data support the application of infrared thermography in a laboratory, clinical and industrial setting for vegetative bacteria, thus may become into an important methodology for the timely and straightforward detection of early‐stage bacterial growth.

## INTRODUCTION

Bacteria are ever present in the environment and in many scenarios have deleterious effects. The food industry, pharmaceutical industry and healthcare are of particular importance, as bacteria can not only spoil products but more importantly lead to morbidity and mortality within populations. Current methods for bacterial detection do not give results in real time, with samples needing to be analysed within laboratories, using a plethora of bacterial culturing and molecular‐based and biochemical‐based detection (Chandler, 2013; Griffith, 2016; Mandal et al., 2011; Salihah et al., 2016; Vu et al., 2014) leading to several emerging technologies for quicker bacterial detection (Behera et al., 2019; Kumar & Ghosh, 2019).

Thermal imaging is a completely safe, noninvasive technology that operates by measuring infrared emission, although its use for bacterial detection is in its infancy. Within the laboratory, *Escherichia coli* in liquid broth has previously been detected by thermal imaging at levels as low as 120 colony forming units (CFU) per ml, albeit in very stringently controlled conditions (Salaimeh et al., 2011); *Staphylococcus aureus*, *Vibrio chloerae*, *Vibrio mimicus*, *Proteus mirabilis* and *Pseudomonas aeruginosa* have also been detected in liquid broth (Lahiri et al., 2012; Salaimeh et al., 2012). In addition, *E*.* coli* has been detected on agar plates using thermal imaging after 11 h of growth under a strict temperature and humidity‐controlled environment (Hahn et al., 2006). Microbial growth has also been detected within soil samples (Kluge et al., 2013) and fungal growth (*Fusarium graminearum* and *F*. *culmorum*) within wheat ears (Al Masri et al., 2017), both within the laboratory. Within an applied setting, the food industry uses thermal imaging to detect foreign bodies in food material (Mohd Khairi et al., 2018; Vadivambal & Jayas, 2011), and within healthcare, thermal imaging has been shown to be able to detect infection in surgical site infections (Childs et al., 2019; Fujita et al., 2013; Siah & Childs, 2015).

In these systems, where bacteria have been detected, it is pertinent to establish the source of the heat being detected. Within the body, the inflammatory phase of wound healing leads to vasodilation and, thus in the increased blood flow, delivers more warm blood to a peripheral wound site. In addition, the presence of bacteria further increases the inflammatory response and thus increases the wound temperature further (Rendell et al., 1997). Bacteria themselves produce small amounts of heat as a product of energy spilling, whereby energy escapes from the proton motivate force (PMF) and is released as heat (Russell, 2007). This heat has been detected by isothermal microcalorimetry (Braissant et al., 2010; Liu et al., 2020) but has not been directly detected in a noninvasive manner. Finally, the presence of bacteria can be seen as the presence of a material with distinct physical properties, and different materials have shown to emit heat in different intensities of infrared radiation (Meola et al., 2004).

The aim of this study was to explore the use of thermal imaging as a method to detect bacteria before their detection by conventional methods in a laboratory setting, thus opening the possibility that thermal imaging may be of use in the healthcare and industrial setting for early detection of bacterial contamination. The source of heat detected, either from the material properties of the bacteria or bacterial metabolism, was determined.

## MATERIAL AND METHODS

### Bacterial strains and growth conditions

Bacterial species were selected based on their prevalence in device‐associated infection. *Escherichia coli* MG1655 (ATCC 47076) (common in catheter‐associated urinary tract infections) was cultured on L broth agar (Lennox, 1955) and *Staphylococcus aureus* SH1000 (derivative of NCTC8325; O’Neil, 2010) (common in cannula‐associated infections) on Mueller Hinton agar (Mueller & Hinton, 1941) in 10 cm petri dishes with 4 mm deep, 12 g/L agar.

For thermal imaging, one half of the agar surface was streaked with bacteria from a single colony using an inoculating loop. The other half of the plate surface was streaked in the same manner with a sterile loop to produce a similar, but bacteria‐free, surface topography to act as an in‐built plate control for thermal imaging. Control plates with no bacteria on either side were streaked with sterile loops to provide an environmental control. Agar plates were then incubated as described at 37℃.

After incubation, the plates were removed individually and placed on a 5 mm layer of insulating material (cotton wool) on the bench. The test area was free from air turbulence (e.g., air conditioning), and the area was free from local sources of heat such as Bunsen burners and extraneous electrical equipment. The thermal camera was positioned on a tripod 0.5 m directly above the plates. The plates were then thermal videoed for 10 s at 20 frames per second.

### Cellular metabolism assays

Agar plates were inoculated as above and incubated at 37℃ for 6 h and removed individually from the incubator, and thermal videos were captured at 10 s. Plates were eradiated with ultraviolet (UV) by treatment at 312 nm for 60 s (plates where in direct contact with UV source: BDH transilluminator, TFX‐20 MX, 100% intensity), and then incubated at 37℃ for a further hour and thermal imaging repeated, controls were put into the UV system with no UV for a comparable time. 2,4‐dinitrophenol (DNP) (10 μl of 0.1 mmol/L or an equal volume of deionized water as a control) was added to eight locations on the streaked section of the plate and the plates incubated at 37℃ for a further 18 h, and the thermal video recording was repeated.

### Thermal imaging and data processing

All thermal video recording was performed using a ThermoVision A40 Infrared Camera (FLIR) paired with ThermaCAM researcher professional 2.9 software (FLIR). The infrared camera had a thermal sensitivity of 0.08℃ and a spectral detection range of 7.5–13 µm, with a −50 to 500℃ temperature detection range. Data processing was performed using MATLAB^©^ R2016a software (MathWorks). Using the MATLAB^©^ graphic interface, the image of a plate was displayed on a computer screen. Then, the *x–y* coordinates of the centre of the plate were determined by clicking on the top, bottom, left and right edges. These coordinates were then entered into a MATLAB^©^ program that calculated the centre of the Petri dish by determining the intersection of the lines joining the vertical and horizontal edges. From the identified centre point, the software then automatically segmented a circular area with diameter 10 cm, avoiding a small area close to the edge of the dish. This area was avoided to reduce any affect the dish itself may have on the measured temperatures. The software then divided the segmented circular area into two equal halves, that is, left (control without bacteria) and right (bacteria) sides. The pixel values within each half of the plate image, representing the temperature in each half, were averaged to obtain two temperature readings for each dish (MATLAB code in supplementary materials). This was repeated for each plate, and an overall average was produced. To determine whether data had a normal distribution, a Kolmogorov–Smirnov test of normality was undertaken, and data did not differ significantly from that which is normally distributed. To determine whether there was a significant difference between the mean temperatures of the left‐ and right‐hand sides of the dishes, Student's *t*‐test was performed.

## RESULTS

### *E. coli* and *S*. *aureus* can be detected by thermal imaging before they are visible under laboratory conditions using conventional imaging

**FIGURE 1:**
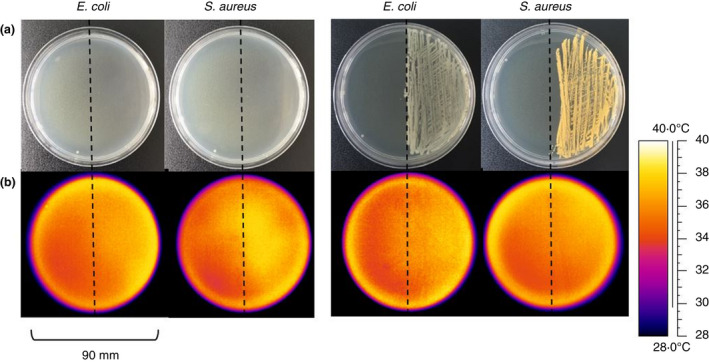
Representative visual and thermal images of agar plates streaked with *Escherichia coli* and *Staphylococcus aureus* at 6 and 24 h of growth. *E. coli* and *S*. *aureus* were streaked onto the right‐hand side of the agar plate and incubated at 37℃ for 24 h with visual (a) and thermal images (b) taken at 6 and 24 h. The dashed line indicates the midline of the plate dividing the plate into the areas with and without the bacterial growth. Thermal images were taken within 30 s of the plates leaving the incubator (*n* = 12)

The use of thermal imaging as a nondestructive method to detect bacterial growth before it can be seen by eye was investigated. *E. coli* and *S*. *aureus* were streaked onto half a petri dishes and incubated for 24 h at 37℃. At the 6 and 24 h time points during this incubation, the plates were removed from the incubator, and conventional and thermal images were taken within 30 s of the petri dishes removal from the incubator (Figure 1).

At 6 h of incubation, neither the *E*. *coli* nor the *S*. *aureus* streak could be seen either by eye or using standard visible spectrum photography, whereas after 24 h of incubation, a thick lawn for both bacteria could be visualized by eye (Figure 1a). At 6 h of incubation, the side of the agar plate inoculated with bacteria (but where the bacterial growth is not yet visible by standard methods) warmer for both *E*. *coli* and *S*. *aureus* (Figure 1b, bright yellow) compared with the agar alone (Figure 1b, orange). In addition, the upper area of the agar plate as shown in Figure 1 is also slightly warmer than the lower area, which is due to the laboratory environment. At 24 h of incubation, the bacterial growth of *E*. *coli* is warmer (Figure 1b, bright yellow) than the agar‐only side of the plate (Figure 1b, orange), whereas there is no obvious difference between the sides of the plate streaked with *S*. *aureus* (Figure 1b, orange). Control plates with no bacteria were also incubated and showed no difference in temperature at 6 or 24 h (data not shown).

**FIGURE 2:**
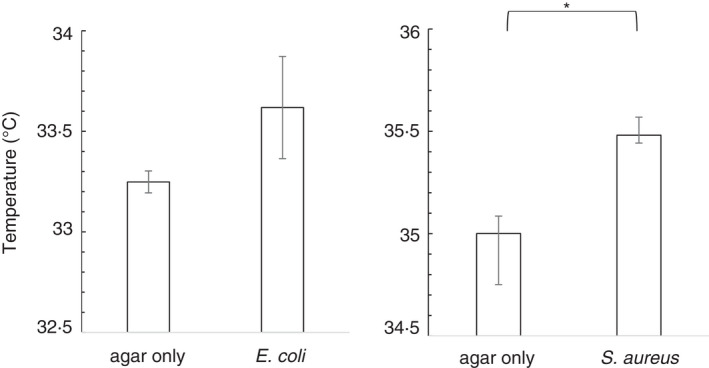
Quantification of thermographs showing bacteria have a statistically significant increase in temperature. Agar plates (12 g/L agar, 4‐mm depth), half streaked with *Escherichia coli* or *Staphylococcus aureus*, were incubated for 6 h at 37℃ and were removed from the incubator, and after 10 s, a thermal video was taken. These were analysed in MATLAB^©^ to determine the average temperature for each side of the plate. Data shown are mean ± standard deviation, *n* = 3; asterisk (*) indicates *p* ≤ 0.05 in a Student's *t*‐test

To quantify the difference in temperature due to bacterial growth, agar plates, with one half streaked with either *E*. *coli* or *S*. *aureus*, were incubated for 6 h, and conventional images and thermal videos taken. The bacterial growth occurred on solid media, and thus, a growth rate was not determined. The thermal imaging was undertaken at 6 h to ensure bacteria have entered the log phase and that early active growth was present. Each thermal image was analysed using MATLAB^©^ to determine the average temperature for each side of the plate, excluding the extreme edge of the plate (Figure 2). The areas inoculated with *S*. *aureus* were statistically significantly warmer than the side of the plate without bacteria. Control plates with no bacteria, but visualized and analysed by the same method, showed no statistically significant difference (data not shown).

### Bacteria emitting heat is a direct result of bacterial metabolism

**FIGURE 3:**
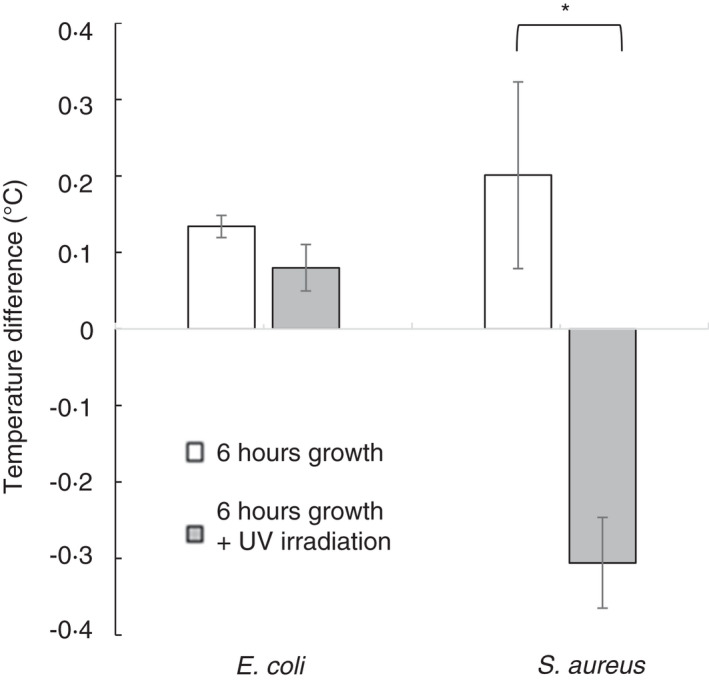
Eradiation with ultraviolet (UV) reduced the increase in heat associated with areas of bacterial growth. Agar plates, half streaked with *Escherichia coli* or *Staphylococcus aureus*, were incubated for 5 h at 37℃, removed from the incubator and irradiated with UV at 312 nm for 60 s. They were returned to 37℃ for a further hour and removed from the incubator, and after 10 s, thermal videos were taken. These were analysed in MATLAB^©^ to determine the average temperature for each side of the plate and the difference between the two determined. Data shown are the mean ± standard deviation, *n* = 3; asterisk (*) indicates *p* ≤ 0.05 in a Student's *t*‐test

To determine if the heat detected from the bacteria was due to the material properties of the bacterial cells, which may have a different rate of cooling from the surrounding agar, or a direct measurement of heat produced by bacterial metabolism, the bacteria were irradiated with UV. Due to the binary nature of this aim, a kill rate of the UV was not determined. Plates were inoculated and grown for 5 h, removed from the incubator and either irradiated with UV or put into the UV system with no UV (control) for a comparable time. All plates were returned to 37℃ for 1 h, before being imaged (Figure 3). UV treatment diminished the heat detected from the area where both *E*. *coli* and *S*. *aureus* had been streaked. *E*. *coli* growth increases heat by 0.13℃ ± 0.02℃ compared with an increase 0.08℃ ± 0.03℃ after UV treatment. *S*. *aureus* growth increases heat by 0.20℃ ± 0.12℃ compared with a decrease of 0.31℃ ± 0.06℃ after UV treatment (*p* < 0.1 and *p* < 0.05 for *E*. *coli* and *S*. *aureus*, respectively). The petri dishes were then incubated for a further 18 h, and no growth was visible on those treated with UV (data not shown).

**FIGURE 4:**
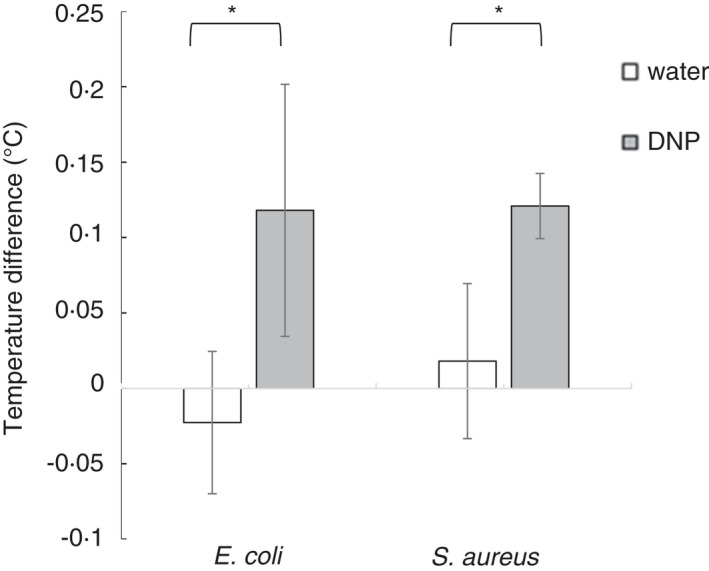
Incubation with 2,4‐dinitrophenol (DNP) increases the heat associated with areas of bacterial growth. Agar plates, half streaked with *E*. *coli* or *S*. *aureus*, were incubated for 5 h at 37℃, removed from the incubator and treated with 10 μl of 0.1 mmol l^−1^ 2,4‐dinitrophenol or deionized water (control). They were returned to 37℃ for a further 18 h and removed from the incubator, and after 10 s, a thermal image was taken. These images were analysed in MATLAB^©^ to determine the average temperature for each side of the plate and the difference between the two determined. Data shown are mean ± standard deviation, *n* = 3; asterisk (*) indicates *p* ≤ 0.05 in a Student's *t*‐test

To further verify that the heat emission detected was directly from bacterial metabolism, DNP was utilized to uncouple the electron transport chain, thus increasing the amount of metabolic energy from the PMF dissipated as heat. Agar was inoculated and incubated for 6 h before DNP or water (control) was added and incubated for a further 18 h (Figure 4). Upon treatment with DNP, the heat associated with bacterial growth increased by more than fourfold in both *E*. *coli* and *S*. *aureus* (*p* < 0.05 for both *E*. *coli* and *S*. *aureus*).

## DISCUSSION

Current nondestructive detection of bacterial contamination on solid surfaces in both clinical and industrial environments is performed using a swab and standard culture method, requiring 18–24 h before viable bacterial colonies are visible to the human eye. Here, infrared thermography is used to detect *E*. *coli* and *S*. *aureus* at 6 h of growth, significantly faster than current nondestructive culture methods. Bacterial agar plates were imaged on an insulating mat. To reduce interference of air movement on thermal images in busy settings, it may be possible to place the bacterial plates in a box, or similar high‐walled container, and is worthy of further investigation in the future. Upon treatment with DNP, the heat associated with bacterial growth increased by more than fourfold in *E*. *coli* and *S*. *aureus* growth. This, taken with the decrease in heat when the bacteria had been killed by heat treatment with UV, indicates that the heat detected is from bacterial metabolism in viable bacterial cells (Cooney et al., 1969). These results indicate that thermal imaging is suitable for detecting metabolically active bacteria under relatively uncontrolled laboratory conditions and, therefore, could applied to the early detection of bacterial growth in a range of applications. The related methodology of isothermal microcalorimetry has been also utilized to detect heat‐emitted bacterial growth (Braissant et al., 2010) and, subsequently, the detection of antimicrobial activity (Liu et al., 2020; Tkhilaishvili et al., 2018). In contrast, however, this work was carried out in a laboratory setting with no bespoke system to control the environment surrounding the experiment nor the requirement to move the bacteria into an isothermal microcalorimeter.

The ability of thermal imaging to detect metabolically active bacteria in situ described here is of note as in a practical setting, the temperature of a sample is likely to also be influenced by the surrounding environment. In addition, the detectable cellular temperature of the bacterial cell will greatly vary in both industrial and clinical environments as it is influenced by factors such as the heat retention ability of the surface on which the bacterial cells are bound. For example, polymethyl methacrylate surfaces which are associated with a high frequency of *E*. *coli* device associated infections and alloy metals which are associated with *S*. *aureus* device associated infections have different thermal properties (Sobolewska et al., 2007). Not all contaminating bacteria are metabolically active, and so thermal imaging may not be suitable for detecting bacterial contamination in all applications. Nonetheless, we believe it will be generally applicable to situations, such as nutrient‐rich environments in the food processing industry and during developing in‐dwelling device‐based infections, where early detection of rapid bacterial growth may avoid serious problems that would otherwise rapidly worsen. The method is statistically robust and does not require specialized temperature control facilities and subsequently may become an important methodology for the timely and straightforward detection of early‐stage bacterial growth.

## CONFLICT OF INTEREST

All authors declare that there is no conflict of interest in this work.

## Supporting information

Supplementary Material
