# Supplementary material for: Infrared thermography can detect previsual bacterial growth in a laboratory setting via metabolic heat detection
Source: J Appl Microbiol. 2021 Jul 24;132(1):2–7. doi: 10.1111/jam.15218 (PMC9292240; doi:10.1111/jam.15218)
Supplement: Supplementary file 1 — Supplementary Material [file JAM-132-2-s001.docx]

**MATLAB code for analysis of thermal images of petri dishes**

clc

X = Insert the X and Y value of the centre of the sample to be analysed.

Y = Insert the diameter of the sample to be analysed.

Z = Insert the file name of the sample to be analysed.

clear all

close all

cx=XXX;

cy=XXX;

diameter=YYY;

load('ZZZ.mat')

I=ZZZ

I=I-273;

minimum= min(min(I));

maximum= max(max(I));

figure(1)

im= imshow(I, [minimum, maximum]);

title('original plate')

figure(2)

subplot(221)

im= imshow(I, [minimum, maximum]);

title('original plate')

radius=round(diameter/2);

ytl=cy-radius;

xtl=cx-radius;

e=imellipse(gca, [xtl, ytl, diameter, diameter]);

mask=createMask(e,im);

mask=mask.*double(I);

minimum= min(min(mask));

maximum= max(max(mask));

figure(2)

subplot(222)

imshow(mask, [minimum, maximum]);

title('selected area')

ytl=cy-radius;

xtl=cx-radius; %

ybl= ytl + 2*radius;

xbl=xtl;

ytr= ytl;

xtr= xtl + 2*radius;

ybr= ytr + 2* radius;

xbr= xtr;

cropt_circle = mask(ytl:ybl,:);

cropt_circle= cropt_circle(:,xtl:xtr);

minimum= min(min(cropt_circle));

maximum= max(max(cropt_circle));

no_of_columns= round(length(cropt_circle)/2);

B=0.2;

left_side= cropt_circle(:, 1:no_of_columns-B*radius);

right_side= cropt_circle(:,no_of_columns+radius*B:2* no_of_columns-1);

min_left=min(min(left_side));

max_left=max(max(left_side));

min_right=min(min(right_side));

max_right=max(max(right_side));

figure(2)

subplot(223)

imshow(left_side, [min_left max_left])

title('selected left side')

subplot(224)

imshow(right_side, [min_right max_right])

title('selected right side')

left_side_signal=[];

right_side_signal=[];

dimension=size(left_side);

threshold=5;

for row=1: dimension(1)

for column=1: dimension(2)

if left_side(row,column)>threshold

left_side_signal=[left_side_signal; left_side(row,column)];

end

if right_side(row,column)>threshold

right_side_signal=[right_side_signal; right_side(row,column)];

end

end;

end;

disp(' ')

disp('-------------------- mean ----------------')

mean_temperature_left_side_of_plate= mean(left_side_signal)

mean_temperature_right_side_of_plate= mean(right_side_signal)

percentage_difference_between_left_and_right_sides_of_the_plate= ((mean_temperature_left_side_of_plate-mean_temperature_right_side_of_plate)/mean_temperature_left_side_of_plate)*100
